# Editorial: Case reports in psychopharmacology, volume III

**DOI:** 10.3389/fpsyt.2025.1721565

**Published:** 2025-11-17

**Authors:** Matej Stuhec, Patricia Di Ciano

**Affiliations:** 1 Faculty of Medicine, University of Maribor, Maribor, Slovenia; 2 Department of Clinical Pharmacy, Ormoz Psychiatric Hospital, Ormoz, Slovenia; 3 Institute for Mental Health Policy Research, Centre for Addiction and Mental Health, Toronto, ON, Canada; 4 Department of Pharmacology and Toxicology, University of Toronto, Toronto, ON, Canada; 5 Dalla Lana School of Public Health, University of Toronto, Toronto, ON, Canada; 6 Campbell Family Mental Health Research Institute, Toronto, ON, Canada

**Keywords:** schizophrenia, mental illness, adverse events, psychopharmacology, pharmacogenetics, antipsychotics, GLP-1 agonist

Mental illness can lead to severe disability and may impact quality of life, even leading to suicidal ideation. The problems associated with mental illness may be more pressing now, as the prevalence of these illnesses may have increased during the recent COVID-19 pandemic (1). Thus, there is a growing need to rise to the challenge to treat these illnesses. This is especially true in view of the fact that many of these patients are treatment-resistant (2). In this third volume of our series of case reports, the overarching theme is the treatment of (mental) illnesses with pharmacotherapies. Continuing from our previous two volumes (3, 4), we present diverse reports on various topics. Not only are there papers on novel treatments for mental illnesses, but we also highlight some new indications for established treatments. We also provide some cautionary tales about the use of some established pharmacotherapies.

The past few years seen a surge in interest in using glucagon-like peptide 1 (GLP-1) receptor agonists. Originally marketed for the treatment of Type 2 diabetes, they are now commonly used for weight management. A paper published this year found evidence that semaglutide may be effective in the treatment of alcohol use disorder (AUD) (5). Consistent with this, Hill et al. report on the successful treatment of AUD with dulaglutide, a GLP-1 receptor agonist. Due to a lapse in insurance coverage, the patient had to discontinue dulaglutide, and they relapsed to previous drinking patterns. Together, this evidence suggests that more large-scale studies are needed into determining the efficacy of GLP-1 receptor agonists for the treatment of AUD and potentially other substance use disorders.

Some mental illnesses can be challenging to manage and two papers in this volume provide some hope for novel methods of managing these disorders. For example, major depressive disorder can be difficult to manage, and many patients may lack the treatment they need. In one case report, Stuhec presents a case of successful treatment of depression by a Pharmacist in primary care. This brings hope that the treatment of depression may extend beyond the physician. Pharmacists collaborated with a general practitioner based on the collaborative practice agreement paper, which the patient also signed. Positive treatment outcomes, such as remission, were reported. This report is in line with the recent developments in Slovenia, where clinical pharmacy services are well developed (6). In another report, on schizophrenia, Hudnik et al. present a case highlighting the role of pharmacogenetics in the treatment efficacy of olanzapine (an atypical antipsychotic, with affinity for serotonin 5-HT2A, dopamine D_2_, histamine H_1_, muscarinic M_1_ and adrenergic α1 receptors) in the management of treatment-resistant schizophrenia. In our previous volume of case reports we also present the utility of pharmacogenetics (4). Thus, novel methods of treatment on the horizon may present new hope for those living with mental illnesses that are difficult to treat.

In the present volume, novel treatment strategies are also presented for two less common illnesses, genital disorder/genitopelvis dysesthesia (PGAD/GPD) and Bainbridge-Ropers syndrome. Rong et al. report on a case of persistent PGAD/GPD that was effectively treated with leuprolide, a synthetic gonadotropin-releasing hormone agonist. The patient exhibited an improvement in genital arousal symptoms as well as a decrease in scores on the Beck Anxiety Inventory and Beck Depression Inventory. Geiser et al. present a potential novel treatment for Bainbridge-Ropers syndrome, typically involving symptomatic treatment. In this report, a 28-year-old male demonstrated an almost complete improvement in challenging behavior following treatment with pregabalin, a modulator of the alpha-2-delta subunit of voltage-gated calcium channels.

Finally, two papers present novel uses of two atypical antipsychotics. Swamy et al. present a literature review and case report highlighting the need to monitor and individually tailor dose adjustments of clozapine (an antagonist at D_2_ dopamine receptors and antagonist at serotonin 5-HT2A receptors) during anti-tuberculosis therapy. This is important because people with schizophrenia are at heightened risk of tuberculosis (7). As well, Goto et al. describe two cases in which aripiprazole (an antagonist at D_2_ dopamine receptors and antagonist at serotonin 5-HT2A receptors, as well as a partial agonist at serotonin 5-HT1A and 5-HT2C receptors) improved auditory abnormal sensations, where traditional approaches such as antidepressants and supportive therapy were insufficient.

Even established interventions can present with new warnings over time. Yao et al. report on an 18-year-old female who developed recurrent acute myasthenia after taking lithium; this resolved after discontinuation. In another study, Zhou et al. suggest that people may develop a dependence on tiletamine (a non-competitive antagonist of the NMDA receptor), a novel psychoactive substance that has emerged in China as an additive to e-cigarettes. This is important in view of some findings that people with mental illness have greater odds of smoking (8). Finally, even commonly-used drugs can manifest with adverse events, as reported by Ahmed et al., where they present the case of a 58-year-old woman who developed bilateral peripheral oedema after low-dose escitalopram for the treatment of major depressive disorder. Complete remission of the oedema was seen within three days.

In sum, this Research Topic presents hope for new treatments of illnesses that can be difficult to manage. Even though great improvement in symptoms can be seen from novel treatment approaches, this Research Topic also highlights the need for continued monitoring of patients undergoing psychotropic treatment.
